# Supplementary material for: Are carbon emissions trading and green financial instruments synergistic? -Comprehensive quantitative research based on content analysis
Source: PLoS One. 2024 Mar 7;19(3):e0298601. doi: 10.1371/journal.pone.0298601 (PMC10919601; doi:10.1371/journal.pone.0298601)
Supplement: S1 Data — (ZIP) [file pone.0298601.s001.zip › Data/地级市Z_F工作报告.zip!/╜¡╦╒/╤╬│╟╩╨/└┤╫╘╣1⁄2╓┌║┼í╛┬φ┐╦╩2╛▌═°í┐_2017.docx]

各位代表:

现在,我代表盐城市人民政府向大会作工作报告,请予审议,并请各位政协委员和其他列席人员提出意见。

本届政府工作回顾

本届政府履职期间,面对复杂的经济环境,我们在省委、省政府和市委的正确领导下,认真贯彻落实党的十八大和十八届三中四中五中六中全会精神,坚持稳中求进工作总基调,适应发展新常态,全面推进“建设新盐城、发展上台阶”,全力以赴稳增长、促改革、调结构、惠民生、防风险,较好完成了“十二五”规划确定的目标任务,实现“十三五”发展良好开局。

经济综合实力明显增强。全市地区生产总值跨越两个千亿元台阶,从2011年的2771亿元增加到2016年的4576亿元,年均增长11.1%;人均地区生产总值跨越三个万元台阶,从38222元增加到63277元,年均增长11.4%;一般公共预算收入年均增长8.3%;固定资产投资年均增长21%;社会消费品零售总额从895亿元增加到1631亿元,年均增长12.8%,消费对经济增长的贡献份额达48.6%。

转型升级取得积极进展。在经济总量持续增长的同时,三次产业结构由15∶47.1∶37.9调整为11.7∶44.8∶43.5。高效设施农业面积增量连续五年全省第一,农业现代化进程苏北第一。汽车、机械、农产品加工等传统产业开票销售达3766亿元。东风悦达起亚公司建成汽车第三工厂,2016年产销汽车突破65万辆。中汽中心盐城汽车试验场建成运营。新能源、节能环保等新兴产业产值年均增长20%以上,成为国家战略性新兴产业区域集聚发展试点城市、国家新能源示范城市、国家新型工业化基地。服务业增加值年均增长12.3%,建成8家省级现代服务业集聚区。高新技术产业产值年均增长27.4%,占工业总产值的比重提高到33.1%,获批国家创新型试点城市、国家知识产权试点城市。

沿海开发开放深入推进。港口建设取得新成效,盐城港“一港四区”建成21个万吨级以上码头泊位,大丰港区和滨海港区10万吨级深水航道建成通航,货物吞吐量连续五年实现千万吨级增长。临港产业加快集聚,新能源淡化海水、国电投协鑫煤电、中粮肉制品深加工、辉山乳业等重大项目相继落户,新能源发电全省领先,海上风电开发建设项目数和规模总量全国第一。港城建设展现新形象,建成一批商住、医院、学校、旅游等功能性配套设施,沿海地区正成为活力最强、增长最快的重要经济板块。平台载体建设取得新突破,中韩盐城产业园成为两国层面的产业合作平台,建成综合保税区,市开发区获批国家生态工业示范园区,盐城高新区获批国家高新区,新增3个省级高新区,与苏南、上海、深圳合作共建的17个园区健康发展,沪苏产业联动集聚区启动建设。五年累计新批外资项目1016个,外贸进出口总额年均增长8.7%。

城乡面貌发生显著变化。中心城市能级提升,以大城市定位纳入长三角城市群规划。大丰撤市建区,形成大市区一主一副、一体化发展新格局。主城区五年实施城建重点项目600多个,完成投资1400亿元,基本建成聚龙湖组团、科教城组团、高教组团,建成内环高架快速路网一期、二期工程,金融城、金鹰天地广场等一批城市功能项目投入使用。推进露水增绿工程,打通串场河两侧滨河道路,建成串场河景观带,市区绿化覆盖率达41.5%,创成国家园林城市、国家卫生城市、全国绿化模范城市。新型城镇化加快推进,全面启动5700公里农村公路提档升级三年行动计划,行政村全部通达客运班车,城乡区域供水基本实现全覆盖,全市常住人口城镇化率从54%提高到61.6%。东台市安丰镇被列为首批全国特色小镇。城乡环境综合整治和生态建设成效显著,市区完成54条骨干河道整治,建制镇污水处理设施覆盖率达95%,成为首批国家生态文明先行示范区。

改革创新激发动力活力。开展国家新型城镇化综合试点。农村土地承包经营权确权登记颁证工作有序推进,联耕联种新模式向全国推广。全市不动产统一登记交易实现市、县两级全覆盖。“放管服”和商事制度改革扎实推进,实行审批项目清单式管理和大厅集中办理、网上办理。大丰区成为全国相对集中行政许可权改革试点单位。“营改增”全面实施,加大财政金融改革创新力度,省市共同设立中韩产业发展基金,政府和社会资本合作成效明显。公立医院改革、机关事业单位养老保险制度改革有序展开。实施“515”人才引进计划,累计引进各类领军人才689名,县县创成省级创业型城市,市区创成省级创业型示范城市。

基础设施建设实现突破。完成交通基础设施建设投入493亿元。“5+1”高速铁路加快推进,盐徐高铁、盐连快铁和盐宁高铁联络线开工建设,盐通、盐泰锡常宜等高铁项目前期工作加快推进,市综合交通枢纽设计方案面向国际征集。阜建高速、盐丰高速建成通车,实现县县通高速目标。南洋机场T2航站楼完成主体施工,年旅客吞吐量突破百万人次。临海高等级公路全线贯通,大丰港疏港航道等一批“通江达海接运河”项目建成通航。完成川东港拓浚和港闸下迁工程,区域排涝减灾和防洪安全保障能力得到新提升。开通互联网国际通信专用通道,建成苏北第一条信息“高速公路”。完成电网建设投资232亿元,新能源消纳和电网可靠供应能力明显增强。

群众生活得到不断改善。实施民生幸福工程,每年兴办20件民生实事。坚持把就业作为民生之本,年均新增城镇就业8万人以上,城镇居民人均可支配收入达到30496元,年均增长9.8%,农村居民人均可支配收入达到17172元,年均增长11%。52万年收入4000元以下的低收入人口如期脱贫,精准扶贫举措得到中央肯定。社会保障水平不断提高,在全国率先建立医保关系异地转移接续机制,推行“四个不出村”服务模式,城乡居民养老保险工作受到国务院表彰。新建中小学、幼儿园170所,新建、改建校舍525万平方米,义务教育发展基本均衡县创建全部通过国家督导评估,义务教育集团化办学工作荣获“全国教育改革创新特别奖”。基本医疗保险、基本公共卫生服务、基本药物制度实现全覆盖,家庭医生签约服务试点工作入选“全国医改十大创新举措”。市三院建成“三甲”医院。实施镇村医疗卫生机构基础设施标准化建设三年行动计划,完成123个基层卫生院、1606个村卫生室基础设施标准化项目。建成了一批文化场馆,创作了一批群众喜闻乐见的文艺精品,淮剧《小镇》荣获国家文华大奖。全市城市社区建成“10分钟体育健身圈”,建成健身步道(自行车道)总长度674公里,完成1802个行政村“村村有体育健身工程”提档升级。完善社会救助体系,率先成立市级社会救助中心,成为全国“救急难”综合试点城市,连续五次获得全国无偿献血先进城市称号。农村敬老院改造全部达到省定“三有三能六达标”标准。获得地方立法权,法治盐城、平安盐城建设深入推进,被表彰为全国社会管理综合治理优秀市。

政府自身建设持续加强。扎实开展群众路线教育实践活动、“三严三实”专题教育和“两学一做”学习教育,严格执行中央“八项规定”和省、市委“十项规定”,“四风”问题得到遏制,“三公”经费大幅下降。推进法治政府、廉洁政府和服务型政府建设,坚持依法决策、科学决策、民主决策,自觉接受市人大及其常委会、市政协以及各民主党派、工商联、社会团体、人民群众的监督,五年办理市人大代表建议915件、市政协委员提案1215件,对人民群众关注的热点问题通过“12345”市民热线等渠道及时给予回应。监察、审计、统计工作得到加强,民族宗教、档案、地方志工作取得新进展,妇女、儿童、青少年、老龄、残疾人、红十字、慈善事业、外事、台侨工作取得新进步。国防动员、人民防空和兵员征集工作不断加强,实现全国双拥模范城“五连冠”。

各位代表,刚刚过去的2016年是“十三五”开局之年。我们按照市委的决策部署,围绕市七届人大六次会议确定的目标任务,统筹推进改革发展稳定各项工作,全市经济社会发展稳中有进、稳中向好,较好完成了年度目标任务。一是经济运行总体平稳。全年地区生产总值同比增长8.9%,规模以上工业增加值增长9.7%,一般公共预算收入完成市人大常委会批准调整方案确定的目标,项主要经济指标增速高于全省平均水平,固定资产投资、服务业增加值、全口径工业开票销售收入等指标增幅位居全省前列。二是改革任务有效落实。扎实推进供给侧结构性改革,积极化解水泥、船舶等过剩产能,坚决打击非法产能。全市商品住宅库存去化周期9.1个月,比上年缩短10个月。新增直接融资381亿元,企业杠杆率有所降低,政府投融资平台资产负债率保持在合理水平。实施减税降费,降低企业成本60亿元。民生保障、公共服务等领域一批补短板项目加快推进。三是转型升级步伐加快。实施产业创新“十大工程”,“两区两城”创新高地建设成效显著,全市新增高新技术企业148家,累计达532家,新认定省级以上企业研发机构45家,清华大学第一个校外国家级实验室落户盐城。累计创成7家省级高端装备制造业特色及示范产业基地,数量位居全省第一。农业发展水平提升,调减低产低效粮田60.4万亩,新增高效设施农业面积16.3万亩,建成各类现代农业园区170个,实现镇级农业园区全覆盖。推进“接二连三”工程,全市农业龙头企业达1661家,农产品出口增长20.9%,个镇、19个行政村被确定为省级电子商务示范镇、村,总量分别居全省第一位、第二位。四是生态建设力度加大。新增绿化造林面积8.8万亩,全省第一。空气优良天数285天,继续保持全省第一。大丰中华麋鹿园创成5A级旅游景区,东台黄海国家森林公园、建湖九龙口国家湿地公园正式挂牌,盐都成为全国休闲农业和乡村旅游示范区。开展“两减六治三提升”专项行动,查处违法企业972家,关闭禁养区养殖场278个,一批环境突出问题得到整治。五是民生实事全面完成。20件为民办实事项目完成投资206.4亿元,比上年增长82.2%。新水源地及跨区域引水工程完成前期拆迁和先导段建设,盐龙湖水厂一期工程加快推进。棚户区改造超额完成年度任务,农村康居工程启动实施。大力引进“名校名师”“名院名医”,17所名校与我市合作办学,建成名院名医工作室44个。完成新一轮精准扶贫建档立卡工作,按年收入6000元新标准实现脱贫10.7万人,26个省定经济薄弱村年集体经营性收入达18万元以上。六是抗灾救灾取得全胜。全力抗击“6·23”特大龙卷风冰雹灾害,以最快速度抢救生命、安置群众、稳定秩序、恢复生产。875名伤员全部得到救治康复出院,39家受损企业6个月内全部复产,抗灾过程中没有发生一起次生灾害、没有发生一起医疗事故、没有发生一起重大负面舆情。灾后重建工作有序推进,20个灾后重建集中安置点已有19个主体封顶,受灾群众生产生活得到切实保障。

来之不易的成绩是全市上下开拓进取、共同奋斗的结果。在此,我谨代表市人民政府,向全市人民、驻盐部队指战员、武警官兵和公安民警,向各位人大代表、政协委员和离退休老同志、社会各界人士,向长期关心、支持盐城发展的海内外朋友,致以崇高的敬意和衷心的感谢!

在总结成绩的同时我们也清醒地看到,当前我市经济社会发展中还存在一些突出问题:产业基础不够厚实,企业创新能力不强,居民收入、企业利润、财政收入“三个口袋”不够充实;资源环境约束趋紧,流域性污染、农业面源污染以及水质性缺水比较严重;民生保障薄弱环节较多,基础设施亟待改善,公共服务资源配置仍不够均衡,扶贫攻坚任务艰巨;政府职能转变还不到位,工作效率不高,不作为、慢作为、庸懒散现象依然存在,干事担当的激励机制有待完善。对这些问题,我们将高度重视,在今后工作中认真加以解决。

今后五年主要目标和2017年工作安排

市第七次党代会确定了如期全面建成小康社会的奋斗目标,描绘了未来五年盐城发展的美好蓝图。书写中国梦盐城篇章,在盐阜大地实现第一个百年目标、开启第二个百年目标新征程的历史使命落在我们身上。全面建成小康社会,是今后五年工作的主旋律。聚焦产业、立足生态、着力富民,是今后五年的发展取向、工作导向和奋斗指向。我们必须清醒认识和准确把握外部环境、内在条件和阶段性要求的新变化,坚持发展第一要务,以强烈的使命感和责任感,不断开辟盐城发展新境界。

做好今后五年政府工作,要全面贯彻以习近平同志为核心的党中央治国理政新理念新思想新战略,认真落实省、市党代会精神和省委省政府以及市委的决策部署,以提高发展质量和效益为中心,以供给侧结构性改革为主线,坚持稳中求进工作总基调,聚力创新、聚焦富民,推进产业强市、生态立市、富民兴市,全面做好稳增长、促改革、调结构、惠民生、优生态、防风险各项工作,确保如期全面建成小康社会,努力建设经济强、百姓富、环境美、社会文明程度高的新盐城。

围绕市第七次党代会确定的奋斗目标,努力实现“三个新提升”:

——聚焦产业强市,经济发展质量实现新提升。经济保持中高速增长,产业迈向中高端,新型工业化、信息化、城镇化、农业现代化和绿色化协同推进,经济发展更多依靠创新驱动。先进制造业和战略性新兴产业规模扩张,传统产业提档升级,现代服务业加快发展,农业现代化水平稳步提升。地区生产总值突破7000亿元,综合经济实力再上新台阶,居民收入、企业利润、财政收入“三个口袋”更加充实。

——坚持生态立市,生态环境质量实现新提升。以改善环境质量为核心的生态文明建设融入全市经济、政治、文化、社会建设各方面和全过程。国家可持续发展实验区建设取得新成效,环境突出问题得到有效治理,源头管控能力不断增强,单位地区生产总值能耗、水耗持续下降,主要污染物排放总量持续削减,重要湿地、河湖水生态系统逐步恢复,数百万亩森林覆盖城乡,人居环境明显改善,盐阜大地天更蓝、地更绿、水更清、空气更清新。

——着力富民兴市,群众生活质量实现新提升。城乡居民收入增长与经济增长保持同步,劳动报酬增长和劳动生产率提高保持同步,居民收入在地区生产总值中的比重逐步提高,“十三五”期间城乡居民人均可支配收入分别达45000元、25000元。保持农村居民收入增幅高于城镇居民收入增幅,人均年收入6000元以下农村低收入人口全部脱贫。覆盖城乡的社会保障体系更加完善,保障水平不断提高,就业、教育、医疗、文化、体育、住房等公共服务体系更加健全,交通出行进入“高铁时代”,饮用水安全得到切实保障,人民群众获得感明显增强,全面小康的成效得到普遍认可。

各位代表,2017年是党的十九大召开之年,也是全面落实省、市党代会部署的开局之年。今年全市经济社会发展的主要预期目标是:地区生产总值增长8.5%,一般公共预算收入同口径增长达到5%的全省平均水平,固定资产投资增长11%,规模以上工业增加值增长9%,全社会研发投入占GDP比重达2.1%,城镇登记失业率控制在4%以内,完成节能减排和大气、水环境质量等各类约束性指标省定年度目标。

重点做好以下八个方面工作:

(一)大力推进转型升级,加快产业强市步伐

推动新兴产业规模化。培育壮大新能源、节能环保、大数据、智能终端、高端装备与新材料等战略性新兴产业,确保全年主营业务收入增长20%以上。推进100个新兴产业重点项目建设,着力构建“新能源+”全产业链,打造新能源及新能源装备制造产业基地,培育千亿级环保产业集群,加快建设国家级大数据产业基地,智能手机产销量突破1000万部。主攻高档数控机床、自动化生产线、智能农机装备,再创一批省级高端装备特色产业基地。

改造提升传统产业。全面落实中国制造2025盐城行动计划,推动汽车、机械、纺织、化工、农产品加工等行业制造装备升级和互联网化提升。组织开展大规模技术改造,推进500项投资超千万元技改项目,实施100项示范推广智能制造项目,工业技改投资增长9%。做强“一部车”,坚持百万辆整车目标不动摇,促进东风悦达起亚公司今年整车销售突破70万辆,加快发展新能源汽车,打造汽车整车、零部件、服务业“三个千亿”产业集群。以创建全国质量强市示范城市为抓手,评定政府质量奖,激励企业弘扬“工匠精神”,开发更多拳头产品。

积极发展现代服务业。实施服务业发展三年行动计划,推动31个市级以上服务业集聚区提档升级,推进50个服务业重点项目加快建设,促进金融服务、现代物流、汽车服务、科技服务等生产性服务业提档加速,生产性服务业增加值占服务业的比重达到41.5%。适应消费升级新趋势,大力发展电子商务、文化创意、旅游休闲、健康养老等新兴服务业,推动实体零售等传统商贸流通业创新转型。

着力培育高新技术企业。把培育高新技术企业作为聚力创新、产业强市的重要抓手,遴选300家科技型企业重点培养,全年新增高新技术企业130家以上。以增强企业自主创新能力、拥有自主知识产权和自主品牌为目标,主动对接大院大所和省“一中心一基地”建设,深入实施知识产权强企行动,新增授权专利8000件以上,新创国家级研发机构1—2家、省级研发机构30家以上。

(二)全力振兴实体经济,促进经济稳定增长

扶持骨干企业加快发展。实施大企业(集团)培育行动,推动100户大企业(集团)通过战略重组、股改上市等方式创新发展,新增15家企业完成股改、20家企业新三板挂牌。健全制度化的政企互动机制和市场化的银企对接机制,继续开展企业“争星创优”活动,对星级企业及企业家给予表彰奖励,优先给予财政扶持。鼓励银行、保险等金融机构加大对实体经济的支持力度,降低制造业企业融资成本,保持制造业信贷合理稳定增长。推进诚信盐城建设,积极营造企业良好发展环境。

培植更多经济增长点。持续扩大有效投资,扎实抓好“两重一实”项目建设,优化投资结构,提高投资效益。开展“产业项目推进年”活动,强化招商引资、招才引智,提高专业化招商水平,围绕建链、补链、强链,着力引进一批龙头型、基地型项目,加快培育一批具有爆发力和引领力的创新型增长点。发挥消费的基础作用,积极培育新型消费业态。增强出口拉动力,促进出口总量扩张、质量提升。

全面落实供给侧结构性改革政策措施。更大力度推动“三去一降一补”,坚决淘汰落后产能,严禁违法违规产能。盘活城市闲置和低效用地,促进产城融合和房地产市场平稳健康发展。加大股权融资力度,提高直接融资比例,推动地方融资平台转型发展,严防政府性债务风险。组织开展“降本增效”暖企行动,进一步降低企业税费负担、制度性交易成本、用能成本、物流成本,确保企业降本60亿元以上。围绕公共服务、基础设施、民生保障、生态建设等薄弱环节和重点领域,继续实施补短板专项工程,确保投资达650亿元以上。

(三)提升农业发展质效,促进农民持续增收

加快调整农业结构。深入推进农业供给侧结构性改革,调减低产低效粮田40万亩,新增立体复合经营30万亩、蔬菜园艺经济作物70万亩、设施农业15万亩;扎实推进沿海百万亩现代渔业产业带建设,着力打造3个10万亩规模渔业产业基地和5个现代渔业园区。大力发展“互联网+”现代农业,培育一批网络化、智能化、精细化的现代农业示范基地。大力实施农业“接二连三”工程,开展百家农业产业化龙头企业提升行动,培植一批规模龙头企业,积极发展外向农业,新增农产品“三品”品牌200个,力争农产品电商交易额突破100亿元。严防严管严控农产品安全风险,加强农业标准化生产,实现农药化肥用量减量化,建设10个农产品质量安全应急快速检测室。

积极发展休闲农业和乡村旅游。组织休闲观光农业“百点创建”行动,争创全国休闲农业与乡村旅游示范县1个,新创省级休闲观光农业示范村3个,新认定市级以上休闲农业示范点20个。鼓励农民通过盘活农村闲置房屋、集体建设用地、林地、湿地等资源,建设精品民宿、自驾游营地等旅游设施。依托珍禽保护区、麋鹿保护区、大洋湾、大纵湖、金沙湖、九龙口、黄河故道等生态旅游资源,加快重点景区建设,高起点规划、高水平建设荷兰花海等一批富有盐城特色的旅游风情小镇,不断完善精品旅游线路,打造中国生态湿地特色旅游目的地。

深化农村各项改革。落实农村土地“三权分置”,全面完成农村土地承包经营权确权登记颁证工作。大力培育家庭农场、农民合作社、农业龙头企业等新型农业经营主体和社会化服务组织,新发展市级以上示范家庭农场50家、培育市级以上农民合作社示范社300家,促进多种形式的适度规模经营。推进农村集体资产产权制度改革,建立“村强带民富”机制,切实保障农民集体收益分配权。全面深化供销社综合改革,发挥好供销社为农服务综合平台作用。

(四)推进沿海开发开放,不断拓展发展空间

构建现代化综合交通网络。加快构建包括高铁、高速、高架等多种运输方式无缝衔接的“一张网”,强力推进“5+1”高速铁路建设,开工建设盐通高铁、市区综合交通枢纽,做好盐泰锡常宜高铁前期工作,完成盐城境内盐宁、盐徐高铁架梁铺轨50%以上,基本建成盐连快铁盐城段主体工程,积极争取纳入京沪高铁新干线规划。推进大丰港区15万吨级深水航道、滨海港区7个5—10万吨级码头泊位、射阳港区5万吨级航道和响水灌河口5万吨级航道整治等工程建设。积极推动淮河入海水道航道工程前期工作。南洋机场T2航站楼建成投运。全力争取规划建设盐宁高速,推动阜建高速南连北延,完成一批国省道干线公路建设年度任务。

加快建设临港新型产业基地。对接长江经济带产业转移和长三角城市群分工协作,推进黄金海岸经济带综合开发和港产城融合发展,争创国家海洋经济发展示范区。组织编制产业发展指导目录,明确各板块产业发展重点,优先发展清洁能源、新能源装备等特色产业,打造沿海先进制造业基地。推进港口物流、海洋生态旅游等产业加快发展,打造一批省级服务业集聚区。

扩大对内对外双向开放。主动融入“一带一路”布局,深化对韩务实合作,加强与欧美和新兴经济体国家经济交往,积极引进利用外资,力争新引进总投资3000万美元以上外资项目50个。与苏南、上海、深圳等发达地区加强区域协作,创建长三角区域产业合作示范区。

着力打造优势载体平台。坚持市本级重抓“两区两城”和中韩盐城产业园、滨海新区,县级重抓省级开发区,镇级重抓专业园区,推进园区特色化、差异化发展,增强要素集聚能力。积极争取上级支持,加快推进中韩盐城产业园一批重点项目建设。充分利用盐田资源,以“急不得”的定力和“慢不得”的进取心,加快建设淮河生态经济带重要门户,市县合力打造滨海新区,创造更好条件,吸引更多战略投资者。实施国家特色产业基地(园区)培育行动计划,争创一批高新技术产业基地、火炬计划产业基地。

(五)加强生态文明建设,塑造独特竞争优势

实施绿色制造行动计划。大力发展“生态+”经济,推动生产方式绿色化转型。严守生态保护、耕地保护、城市开发边界“三条红线”,严把“产业导向、项目准入、节能减排”三个关口,提高准入门槛,强化源头管控,实行最严格的环境保护、水资源管理、节约用地和耕地保护制度,确保万元GDP能耗下降到0.49吨标煤、水资源消耗下降到108立方米,亿元GDP占用建设用地面积下降到833亩,自然湿地保护率提高到44%。

全面整治环境突出问题。认真抓好中央环保督察问题整改落实,全面推进“263”专项行动,坚决打赢治气、治水、治土三大攻坚战。实施新一轮化工行业专项整治,关停重组淘汰小化工企业30家以上。齐抓共管、合力推进空气质量提升工程,确保空气质量全省第一、全国前列。扎实抓好水环境整治、城乡垃圾整治,控制畜禽养殖、农业面源污染,全市地表水水质达到国家、省考核要求。开展城乡污水治理攻坚,推进市区和县城建成区黑臭水体治理,实施市区核心区64条(段)、84公里河道整治。

深入开展生态创建活动。推进大规模国土绿化,实施沿海百万亩生态防护林建设工程,为子孙后代再添“一片林”,今年新增成片造林10万亩,改造提升成片林15.3万亩。新建完善城镇绿地2600公顷,推进铁路、公路、河道沿线绿色景观廊道建设,打造一批森林小镇和森林村庄。依托两个国家级自然保护区打造生态特区,加大国家湿地公园、国家森林公园创建力度,积极申报世界自然遗产,争创更具影响力的生态品牌。

创新生态文明体制机制。深入推进国家可持续发展实验区综合配套改革,加快建设国家生态文明建设示范市。认真落实河长制,完善市县镇村四级河长体系,健全工作机制,强化考核问责。实施环保机构监测监察执法垂直管理改革,全面推行排污权有偿使用和交易制度,开展自然资源统一登记试点,推进领导干部自然资源资产离任审计,加大环境违法行为查处力度,不断完善环境保护长效机制。

(六)坚持城乡一体建设,扎实推进新型城镇化

推进中心城市建设。按照“精明增长”“紧凑城市”理念,坚持组团式发展,以人为本建设“一座城”,重点推进小新河以南新型复合功能组团、范公堤高铁综合交通枢纽组团、大洋湾生态组团、先锋岛生态组团、斗龙港生态组团规划建设,打造一座智慧、人文、生态、宜居的江苏沿海现代化中心城市。充分挖掘“河、堤、湾、岛、港”的历史底蕴和生态资源,聘请一流规划单位,做优城市设计,推进“多规合一”,以高水平规划引领各组团建设,年内启动实施一批基础设施、公共配套项目,完成投资63亿元。根据城市总体规划确定的重点向南向东发展方针,统筹推进通榆河西城中、城南、城西、城北四个片区开发,向小新河以南拓展新区,同时进一步完善通榆河东城市功能配套,促进产城融合、职住平衡。以改造提升建军路核心商圈为重点,有序推进棚户区、城中村成片改造,主城区完成棚户区改造3500户和9个老旧小区整治,不断改善老城区人居环境。

提升城市发展品质。优化产业、居住、休闲等功能和空间布局,完善教育、医疗、文化、体育等公共服务设施。实施S331省道东延等道路工程,开通S226省道东线公交,加快盐丰一体化。建设市区内环高架三期工程,推进人民路等主干道快速化改造,打通腾飞路,实施东进路东延、黄海路东延等项目,进一步完善市区道路系统。实施内环高架沿线绿化及景观带建设、串场河景观带二期工程,加强城市环境综合整治,加快静脉产业园建设,尽快启动垃圾发电厂搬迁。推进城市管理体制改革,开展生态园林城市、森林城市、海绵城市、智慧城市建设,积极发展绿色建筑,加强城市地下综合管廊、防洪工程、污水管网完善和雨污分流改造等基础设施建设,争创全国文明城市、国家森林城市、国际花园城市和联合国人居环境奖。

促进城乡区域协调发展。进一步优化城镇布局,完善“中心城市—县(市)城—重点中心镇—新型农村社区”四级城镇体系,促进联动发展,放大中心城市辐射带动效应。强化县城县域中心功能,加快产业发展,促进人口集聚。推进特色小镇建设,繁荣壮大镇村经济,将13个全国重点镇、个省级重点镇和30个重点中心镇培育成工业强镇、商业重镇或旅游名镇。深入推进“美丽乡村”建设,新培育省、市级“美丽乡村”40个、“康居村庄”160个,创建新型农村示范社区30个。深入实施国家新型城镇化试点,创新户籍、土地、财政、住房等政策举措,落实农业转移人口市民化、投融资机制多元化、产城融合发展等重点任务。

(七)推动社会事业发展,优化公共服务供给

办好人民满意教育。围绕促进教育公平和提高办学质量,进一步优化教育布局,新建中小学、幼儿园60所以上,逐步解决部分学校班额规模过大问题,促进更多义务教育学校达到省定标准化办学要求。积极支持和规范民办教育发展,推进与名校合作办学,引进更多优质教育资源,提高办学质量。深化职业教育产教融合,支持职教联盟集团化办学和现代职业教育体系建设,支持在盐高校硕士学位授予点和重点学科建设,加快建设一流应用型大学。

打造“健康盐城”。推进医药卫生体制改革,加强分级诊疗制度和医联体建设,加快建立现代医院管理制度。推进市一院、城南医院一体化管理,实施市中医院、市妇幼保健院改扩建工程,完成34个基层卫生院标准化建设任务。加大医疗专家的培养与引进力度,深化对外合作办医,新增10个名医工作室,鼓励和支持基层医疗机构灵活运用多种方式招聘、留用重点岗位急需人才。加强计划生育服务管理,提高妇幼健康服务水平。广泛开展全民健身运动,主城区新建健身步道55公里,对50个社区75处体育设施提档升级。做好老龄、老干部和关心下一代工作,建立健全多层次社会化养老服务体系,关爱农村留守老人、留守妇女、留守儿童,切实保障妇女和未成年人权益。继续支持残疾人和慈善公益事业发展。

促进文化繁荣。积极培育和践行社会主义核心价值观,宣传“盐城好人”事迹,不断提升市民文明素质和社会文明程度。推进建设和改造提升一批文化重点项目,新建500个基层综合性文化服务中心,推动基本公共文化服务标准化、均等化。实施文化惠民工程,推进“书香盐城”建设。实施文艺精品创作工程,加强淮剧、杂技等专业院团建设,做好文化遗产传承保护工作。

(八)切实保障改善民生,着力推进富民兴市

多措并举促进居民增收。完善持续提高城乡居民收入水平的政策措施,推动富民增收与经济增长互促共进。大力推进创业富民,落实创业财税优惠政策,加大金融支持力度,鼓励返乡下乡人员创业。实施积极的就业政策,着力拓展就业空间,强化职业技能培训,新增城镇就业9万人。实施技能人才、新型职业农民、科研人员、高校毕业生等八类重点群体收入激励政策,激发重点群体活力,带动城乡居民增收。完善收入分配制度,健全职工工资正常增长机制,逐步提高最低工资标准。推进精准扶贫脱贫,完成14万建档立卡低收入人口脱贫任务,促进省定35个经济薄弱村年集体经营性收入达到18万元以上,更高水平实现新“八有”目标。

提高社会保障水平。整合城乡基本医疗保险制度,实施全民参保登记计划,推进基本医疗保险跨省异地就医联网结算,逐步提高离退休人员基本养老金水平、城乡居民基本养老保险基础养老金标准和基本医疗保险财政补贴标准。实施民生救助“三项保险”,推进各项救助制度衔接。逐步提升城乡低保、特困人员供养、孤儿养育标准,切实保障低收入群体基本生活。推进廉租住房和公共租赁住房统筹建设、并轨运行,大力实施货币化住房保障,解决困难群众基本住房需求。

继续办好民生实事。聚焦就业服务、社会保障、终身教育、医疗健康、生态环境、公众出行等公共服务体系建设,实施创业富民“四送”工程、市民“一卡通”工程等20件实事,完成投资93亿元,确保当年见效。市县联动抓好引水、治水、修路、建房,基本建成新水源地及跨区域引水工程,让惠及近500万人口的“一桶水”早日流向千家万户,确保完成农村公路提档升级工程、城乡河道综合整治工程和农村康居工程年度目标任务。加快推进“6·23”灾后重建工作,确保今年6月底前全部完成。

维护社会和谐稳定。深入开展“七五”普法,提高全民法治意识。创新社会治理,推进平安盐城、法治盐城建设。持续推进社区“减负增效”,实现镇及街道“政社互动”全覆盖。坚守生产安全和公共安全底线,完善社会矛盾排查预警和调处化解机制、突发事件应急处置联动机制,突出抓好危险化学品等重点领域安全生产专项整治,坚决防范重特大事故发生。加强食品药品专项整治和综合治理,升级立体化信息化社会治安防控体系,严厉打击各类违法犯罪行为。支持、服务国防和军队改革,推进军民融合深度发展,开展双拥共建活动,巩固和发展军政军民团结。

建设人民满意政府

如期实现全面建成小康社会目标,对政府自身建设提出了更高要求。新一届政府将牢固树立以人民为中心的发展思想,切实增强政治意识、大局意识、核心意识、看齐意识,严守政治纪律和政治规矩,提振干事创业精气神,努力建设人民满意的服务型政府。

致力改革创新,做好社会期待政府做的事。进一步转变政府职能,厘清政府与市场边界,充分发挥市场在资源配置中的决定性作用,激发市场主体活力。坚持“亲”的服务,坚守“清”的规则,构建健康政商关系和优良营商环境。加快建立以权责清单为核心的阳光高效审批体制,深入推进“放管服”改革,依法编制政府权力清单、部门责任清单、行政审批目录清单和事中事后监管清单,推进清单管理动态化、标准化、透明化。按照“一站式服务、一次性办成”的要求,推动全市“互联网+政务服务”建设,促进实体大厅与网上平台线上线下融合,完善政务服务平台,让企业和群众少跑腿、不跑腿就能办成事。推进企业投资审批市县同权、扁平化管理,进一步优化审批流程。深化商事制度改革,推行工商注册便利化。着力营造创新环境,积极推进以科技创新为核心的全面创新,把政策、资金、人才、技术叠加在一起,为创新创业者提供更好的工作空间、网络空间和资源共享空间。

提升行政效能,做好政府自身应当做的事。谋定后动、分级负责,提高政府工作组织化程度,做到“党委有决策,政府就行动,落实见成效”。集中精力重抓“一张网、一桶水、一部车、一片林、一座城”,办好这些打基础利长远的大事。全力推进重大项目、重点工程和民生实事,明确各级各部门领导成员和各个板块、条线工作责任,建立“单月会办、分级会办,双月督查、分级督查”推进机制,确保项目建设进度。大力弘扬求真务实作风,说实话办实事求实效,进一步提高政府的执行力和公信力。建立五星级企业市长“服务直通车”、四星级企业县长“服务直通车”制度,确保向企业承诺的事马上就办、办好办成。开展“学先进、找差距,补短板、促发展”活动,引导各地各部门提高工作标准,提升专业水平,创造特色,打造亮点,争当全国全省典型示范。建立容错纠错机制,强化正向激励,激发担当精神,营造想干事、敢干事、干成事的浓厚氛围。

坚持依法行政,做好法律规定必须做的事。恪守法无授权不可为和法定职责必须为的原则,建设法治政府。推进政府工作人员尊法学法守法用法,增强运用法治思维和法治方式处理政府事务的能力,健全依法决策机制,落实重大行政决策法定程序和第三方评估制度,实现政府法律顾问制度全覆盖。积极推动城乡建设与管理、环境保护、历史文化保护等领域的地方立法,规范立法程序,提高立法质量。抓好政务公开和政府信息公开,推动各县(市、区)政府全面开展行政复议委员会试点工作。自觉接受人大及其常委会的法律监督和人民政协的民主监督,虚心接受社会和舆论的监督。执行问责条例,加大问责力度,坚决纠正乱作为、不作为、慢作为,切实规范行政行为。

加强廉政建设,坚决不做违反纪律规矩的事。严格执行中央“八项规定”、国务院“约法三章”和省、市委“十项规定”,紧盯老问题,关注新动向,坚决防止“四风”反弹回潮。严格落实厉行节约反对浪费制度规定,政府带头过“紧日子”,严控“三公”经费支出。落实政府系统党风廉政建设责任制,强化“一岗双责”,加快形成不敢腐、不能腐、不想腐的体制机制。加大行政监察和审计监督力度,加强对公共资金、公共资源交易、国有资产资本的监管,坚决查处腐败行为。坚定理想信念,弘扬铁军精神,着力打造一支政治坚定、业务精良、作风过硬、清正廉洁的高素质公务员队伍。

各位代表!做好今后五年以及今年的工作,任务艰巨,使命光荣。让我们紧密团结在以习近平同志为核心的党中央周围,在省委省政府和市委的坚强领导下,攻坚克难,砥砺前行,以推进全面小康社会建设的优异成绩,迎接党的十九大胜利召开。
